# Hygroscopic motions of fossil conifer cones

**DOI:** 10.1038/srep40302

**Published:** 2017-01-11

**Authors:** Simon Poppinga, Nikolaus Nestle, Andrea Šandor, Bruno Reible, Tom Masselter, Bernd Bruchmann, Thomas Speck

**Affiliations:** 1 Plant Biomechanics Group, Botanic Garden, University of Freiburg, Faculty of Biology, D-79104 Freiburg im Breisgau, Germany; 2 Freiburg Materials Research Center (FMF), University of Freiburg, D-79104 Freiburg im Breisgau, Germany; 3 BASF SE Advanced Materials and Systems Research, D-67056 Ludwigshafen, Germany; 4 Department of Orthopedics and Traumatology Heidelberg, Heidelberg University Hospital, D- 69118 Heidelberg, Germany.

## Abstract

Conifer cones represent natural, woody compliant structures which move their scales as passive responses to changes in environmental humidity. Here we report on water-driven opening and closing motions in coalified conifer cones from the Eemian Interglacial (approx. 126,000–113,000 years BP) and from the Middle Miocene (approx. 16.5 to 11.5 million years BP). These cones represent by far the oldest documented evidence of plant parts showing full functionality of such passive hydraulically actuated motion. The functional resilience of these structures is far beyond the biological purpose of seed dispersal and protection and is because of a low level of mineralization of the fossils. Our analysis emphasizes the functional-morphological integrity of these biological compliant mechanisms which, in addition to their biological fascination, are potentially also role models for resilient and maintenance-free biomimetic applications (e.g., adaptive and autonomously moving structures including passive hydraulic actuators).

The capability of wood and woody materials to absorb and desorb water, and hence to swell or shrink relies on the chemical composition and microstructure^1^. Mainly hemicelluloses and amorphous cellulose bind water in the cell walls and cause hygroscopic swelling or shrinkage of woody materials. Because physical and chemical factors (caused by environmental influences) and microorganisms (fungi, bacteria) lead to deterioration of the structural integrity over time and, therefore, result in irreversible changes in the physical, chemical and mechanical properties, it is not surprising that hygroscopic behavior of wood is up to now only reported from samples of a maximum age of ~1300 years^2^.

The opening and closing of woody conifer cones, a phenomenon known in various taxa such as pines and cypresses, is also governed by a hygroscopic swelling/shrinking mechanism. On each pine cone, dozens of seed scales are arranged in a Fibonacci sequence^3^ and move as a passive reaction to changes in humidity, as dictated by their structural setup (i.e., their functional morphology)^4^,^5^. Under wet conditions (which is unfavourable for wind dispersal), the pine scales are curved upwards, the cone is closed and the winged, airborne seeds are protected and hindered from being shed. Under dry conditions, however, the scales bend down and liberate the seeds, which subsequently fly away. Pine scales, as seen in longitudinal section, are constructed as functional bilayers with a highly swellable, lower sclereid layer (with cells which are ~20% longer in their wet state as compared to their dry state) and a considerably less swellable upper layer composed of sclerenchymatous strands embedded in a matrix of ‘brown tissue’^4^,^5^,^6^,^7^. Based on the different swelling and shrinking properties of these tissues the scale reacts similarly to a bimetallic strip^8^, but responds to humidity changes instead of heat.

Mature pine cones are shed and decompose on the ground^9^. This could be one of the reasons why the reproductive organs of the genus *Pinus* and, especially, three-dimensionally preserved macrofossils of cones are very rare in the fossil record^10^. Until now it remained unclear if such ancient structures can still move hygroscopically, i.e. as to whether their structural-functional integrity has been conserved over the course of time. Respective biophysical and functional-morphological investigations of fossil plant material may be conducted to shed light on the evolution of movement phenomena in plants, as well as on design principles of functionally highly resilient structures. The results from these studies could then be used in biomimetic approaches, e.g. for developing bending-active technical structures and autonomous actuators with low maintenance requirements. The paleontological collection of the Freiburg Botanical Garden houses three well-preserved ancient conifer cones. *Pinus* sp. 1 (Eemian Interglacial, approx. 126,000–113,000 years BP, Tarantian, Late Pleistocene) was found in 1965 in the ‘Else’ mine on Lüneburg Heath, Germany, and was initially embedded in diatomaceous earth (‘Kieselgur’). The much older *Pinus* sp. 2 and *Keteleeria* sp. specimens from the Middle Miocene (approx. 16.5 to 11.5 million years BP) were found in 1963 in a surface brown coal mine in the area of Frechen, Germany (Fig. 1a–c). We tested if the coalified structures of the fossil *Pinus* and *Keteleeria* cones and scales were still capable of passive hydraulic motions as in their extant relatives. For comparison, a freshly fallen, small Scots Pine (*P. sylvestris*) cone was also tested; this species was selected for comparison because of similar dimensions to the fossil cones.

## Results and Discussion

**Figure 1:**
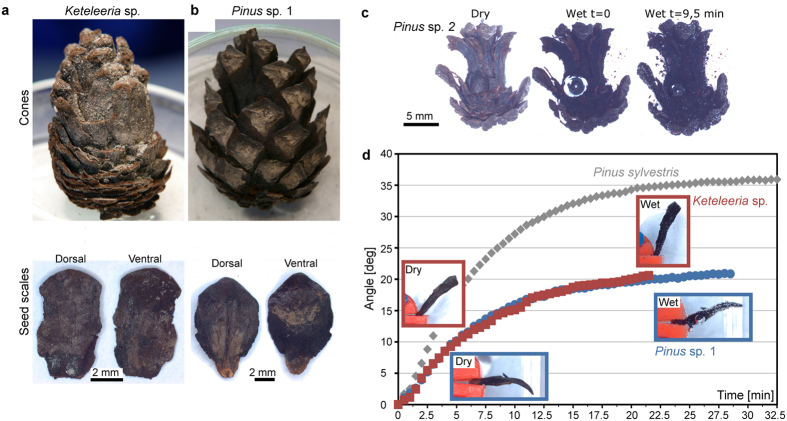
Passive hydraulic actuation of the coalified cones and, for comparison, of a cone scale of extant *P. sylvestris*. (**a**) Cones and scales of *Keteleeria* spec. from the Middle Miocene and (**b**) of *Pinus* spec. 1 from the Eemian Interglacial. (**c**) Hydration of the whole *Pinus* spec. 2 cone from the Middle Miocene, the timescale after wetting is indicated. At t = 9,5 min, the swelling process is completed and the seed scales are notably bent upwards. Frames are from Supplementary Video S1. (**d**) Hydration and angular change over time of coalified, separated seed scales (red: *Keteleeria* sp., blue: *Pinus* sp. 1, grey: *P. sylvestris*).

**Figure 2:**
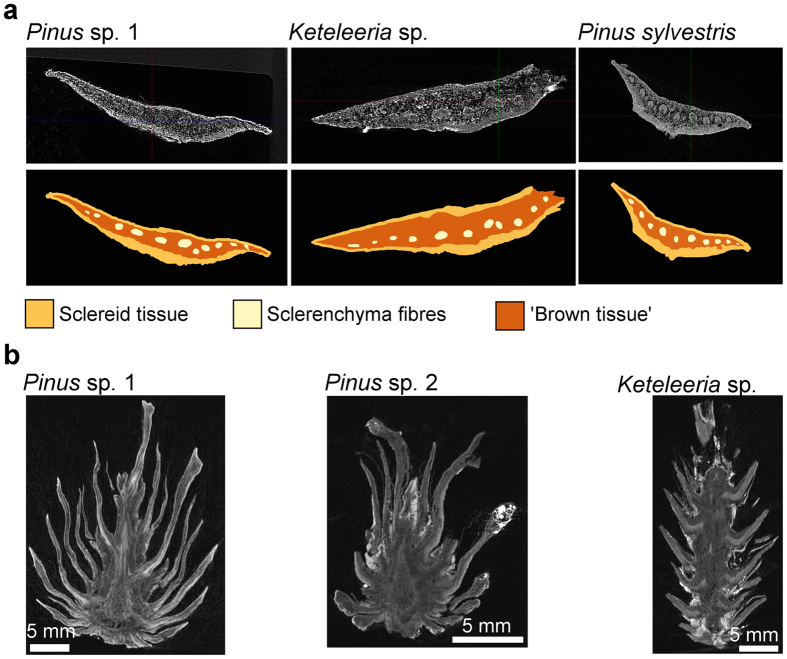
Functional morphology of the coalified cone scales and, for comparison, of a cone scale of extant *P. sylvestris*. (**a**) Cross sectional μCT images (upper row) and schematic representation (lower row) of scales (left: *Pinus* sp. 1, middle: *Keteleeria* sp., right: *Pinus sylvestris*). (**b**) Longitudinal sectional μCT images of the whole three fossil cones, indicating low levels of mineralization inside most of the pine cone tissue and a few strongly absorbing, mineralized spots located mainly on the outside of the samples. Brightness and contrast have been adjusted in the μCT images for clarity.

Our investigations demonstrated that the whole *Pinus* sp. 2 cone (Fig. 1c, Supplementary Video S1), as well as separated seed scales of *Pinus* sp. 1 and *Keteleeria* sp. (Fig. 1d) showed distinct bending movements after being transferred from a very dry environment into a container filled with tap water. The increase of scale mass during hydration (~1.6 fold in *Pinus* sp. 1 and ~2.1 fold in *Keteleeria* sp.) was in the same range as in a similarly sized scale of *P. sylvestris* (~1.8 fold) (Table 1). The extent of angular change of the cone scales over time (=bending deformation), however, is smaller for the fossil taxa, probably due to structural deterioration or delamination of the swelling/shrinking tissues that dictate the movement of the scales. X-ray micro-CT studies of single scales (Fig. 2a) reveal a structural bilayer setup as seen in scales of extant pines, with well visible sclereid layers, sclerenchymatous strands and surrounding matrices. The X-ray absorption of most of the scale tissue was found to be in a similar range as for the recent pine cone scale specimens tested for comparison. This suggests that only minor mineralization effects have occurred in the tissues of the fossilized cone scales. Scans of entire cones (Fig. 2b) indicated that mineralization was low almost throughout (cf. ref. 11), with only a few spots of strongly absorbing mineral material present, suggesting that the observed capability for hygroscopic movement most presumably has been conserved by the apparently dominant process of coalification.

This is the first report of passive-hydraulic actuation in plant organs that are millions of years old, revealing the exceeding resilience of such mechanical systems. Nature impressively demonstrates that it can produce compliant mechanisms which by far outperform man-made movable flaps in terms of long-time and maintenance-free functionality, raising the bar to a new level for current research on the biomimetic transfer of plant movement principles into movable technical systems^12^,^13^,^14^,^15^,^16^,^17^.

## Methods

We carefully separated one seed scale from each of the fossil cones of *Pinus* sp. 1 and *Keteleeria* sp. with a forceps. Separation of scales from the *Pinus* sp. 2 cone was not possible. The scales and the cone were stored in a drying oven (60 °C, 8 h) (TK/L 4250, Ehret Labor- und Pharmatechnik, Emmendingen, Germany) to ensure complete dryness. The scales were then quickly weighed with an analytical balance (ABT 220-5DM, Kern & Sohn GmbH, Balingen-Frommern, Germany) and transferred individually into a container where they were held with a small plastic clamp. The dried cone was placed on putty to avoid rolling. The container was then immediately and carefully flooded with tap water, and the motions were recorded using a SZX9 stereo microscope (Olympus Corp., Tokyo, Japan) and a PixeLINK (Ottawa, Canada) USB 3.0 camera (recording speed: one frame per 30 seconds). After the scales ceased to move, they were carefully dabbed dry and weighed again. For comparison, the same procedures were conducted with a scale from a present-day *P. sylvestris* cone that had recently fallen off a tree growing in the Botanic Garden Freiburg and showed no visible signs of decomposition.

μCT analyses: μCT scanning was performed on a Bruker Skyscan 1172 μCT system (Bruker Skyscan, Antwerp, Belgium) equipped with a 10 W X-ray generator with a maximum acceleration voltage of 100 kV (Hamamatsu, Hamamatsu Japan) and an 11 MPixel X-ray camera (Ximea, Münster, Germany). Imaging of the cone scales was carried out with unfiltered X-rays of 40 keV energy with a spatial resolution of 3.99 μm. In order to avoid actuation effects caused by moisture changes during the measurement, the scales were kept in PE tubes of 12 mm diameter and gently fixed with low-density Basotect™ foam (BASF SE, Ludwigshafen, Germany) to avoid mechanically induced movements of the scales as well. Imaging of the whole cones at equilibrium moisture was performed on a Bruker Skyscan 1076 small animal μCT system with a pixel resolution of either 17.7 μm or 35.4 μm. The X-ray energy used was again 40 keV.

## Additional Information

**How to cite this article**: Poppinga, S. *et al*. Hygroscopic motions of fossil conifer cones. *Sci. Rep.* **7**, 40302; doi: 10.1038/srep40302 (2017).

**Publisher's note:** Springer Nature remains neutral with regard to jurisdictional claims in published maps and institutional affiliations.

## Supplementary Material

Supplementary Video S1

Supplementary Information

## Figures and Tables

**Table 1:** Scale masses.

Specimen	Mass (dry) [g]	Mass (wet) [g]
*Pinus* sp. 1	0.01945	0.03015
*Keteleeria* sp.	0.03722	0.07842
*Pinus sylvestris*	0.02971	0.05294

The masses of the observed wet and dry scales.
