# Supplementary material for: Hygroscopic motions of fossil conifer cones
Source: Sci Rep. 2017 Jan 11;7:40302. doi: 10.1038/srep40302 (PMC5225473; doi:10.1038/srep40302)
Supplement: Supplementary Information [file srep40302-s2.pdf]

Supplementary Information for

Hygroscopic motions of fossil conifer cones

Simon Poppinga,[1,2,3*] Nikolaus Nestle,[4] Andrea Šandor,[4] Bruno Reible,[5] Tom Masselter,[1] Bernd Bruchmann,[3,4] Thomas Speck[1,2]

[1]Plant Biomechanics Group, Botanic Garden, University of Freiburg, Faculty of Biology, D-79104 Freiburg im Breisgau, Germany.

[2]Freiburg Materials Research Center (FMF), University of Freiburg, D-79104 Freiburg im Breisgau, Germany.

[3]Joint Research Network on Advanced Materials and Systems (JONAS).

[4]BASF SE Advanced Materials and Systems Research, D-67056 Ludwigshafen, Germany.

[5]Department of Orthopedics and Traumatology Heidelberg, Heidelberg University Hospital, D-69118 Heidelberg.

[*]Corresponding author. Email: simon.poppinga@biologie.uni-freiburg.de

Supplementary Video S1: Time-lapse recording of the hydration motion of the *Pinus* sp. 2 cone (recording speed: 1 frame per 30 seconds, playback speed: 20 fps).
